# (Not) “just your nature”. Diagnostic journeys of adults with neurofibromatosis type 1 in Poland

**DOI:** 10.3389/fgene.2025.1713304

**Published:** 2026-01-07

**Authors:** Katarzyna Kowal, Jan Domaradzki

**Affiliations:** 1 Wladyslaw Bieganski Collegium Medicum, Jan Dlugosz University in Czestochowa, Czestochowa, Poland; 2 Department of Social Sciences and Humanities, Poznan University of Medical Sciences, Poznan, Poland

**Keywords:** diagnostic journey, diagnostic odyssey, healthcare navigation, neurofibromatosis type 1, patient experiences, rare disease, uncertainty

## Abstract

**Background:**

Neurofibromatosis type 1 (NF1) is a rare autosomal dominant disorder that presents complex physical, emotional, and social challenges. Despite clear diagnostic criteria, NF1 is often diagnosed late due to vague early symptoms, everyday or non-medical interpretations of early signs, and limited access to specialists and genetic testing. This study explores how individuals with NF1 experience the diagnostic process in this context.

**Methods:**

Ninety-three adults with medically confirmed NF1 participated in in-depth interviews conducted between August 1 and 31 December 2020. Data were analyzed using reflexive thematic analysis, following Braun and Clarke’s six-phase framework to explore patterns in participants’ experiences and meaning-making related to diagnosis.

**Results:**

The diagnostic pathway for NF1 was often prolonged, fragmented, and emotionally taxing. Delays frequently stemmed from the normalization of early signs (e.g., café-au-lait macules or subcutaneous nodules) by both families and physicians. Many participants received their diagnosis incidentally, or only after persistent self-advocacy, parental initiative, or the diagnosis of their child. While some family members, particularly mothers, played an active diagnostic role, others responded with denial, minimization, or reluctance to discuss symptoms, which in turn prolonged the search for answers. Structural factors, such as limited NF1-specific familiarity among primary care providers, constrained referral pathways, and regional disparities, further complicated the diagnostic odyssey. The unpredictability and burden of diagnosis often led to frustration and emotional fatigue, particularly among those who lacked a family history of the disease.

**Conclusion:**

This study emphasizes the need for earlier NF1 recognition through enhanced clinician awareness, better diagnostic coordination, and access to genetic and psychosocial support. It also highlights the role of patient agency and the emotional cost of delayed diagnosis in rare diseases. These findings point to several actionable priorities, including the need to strengthen NF1-specific training for primary care physicians, implement clearer referral pathways, and expand access to genetic counselling and psychosocial support.

## Introduction

Neurofibromatosis type 1 (NF1), also known as von Recklinghausen syndrome (ICD-10: Q85.0; ICD-11: LD2D.10; OMIM: 162200, 162210, 613675; UMLS: C0027831; MeSH: D009456), is a rare, complex, and multisystem neurocutaneous disorder caused by mutations in the NF1 tumour suppressor gene located on chromosome 17q11.2 (Garcia et al., 2022). It is classified among RASopathies, a group of conditions resulting from mutations in the RAS-MAPK signalling pathway, which regulates cell growth (Lau et al., 2000; Anastasaki et al., 2022). These mutations lead to an elevated risk of both benign tumours (e.g., neurofibromas) and malignancies, including breast cancer, leukaemia, and sarcomas such as malignant peripheral nerve sheath tumors (MPNST).

NF1 is one of the three major neurofibromatoses, alongside neurofibromatosis type 2 (NF2) and schwannomatosis. NF2 is primarily characterised by bilateral vestibular schwannomas and progressive hearing loss, whereas schwannomatosis involves multiple painful schwannomas without the hallmark cutaneous features of NF1 (Tamura, 2021). Although these conditions share certain neurocutaneous manifestations, they are distinct clinical entities, each with its own genetic basis, tumour profile, and management pathway. In this study, we focus exclusively on NF1 because it is the most common and phenotypically variable form, associated with a broad spectrum of cutaneous, neurological, skeletal, and cognitive manifestations, as well as some of the most substantial diagnostic challenges and psychosocial consequences.

NF1 follows an autosomal dominant inheritance pattern, with a 50% chance of being inherited, although approximately half of the cases arise from *de novo* mutations (Legius and Brems, 2020). The global prevalence of NF1 is estimated at 1 in 1,900 to 3,500 individuals (Kallionpää et al., 2018; Iheanacho et al., 2022; Lee et al., 2023), with 12,000 to 15,000 individuals affected in Poland (Karwacki et al., 2021; Karwacki, 2025).

Disease severity can vary widely. The Riccardi severity scale categorizes NF1 into four levels, from minimal to severe involvement (Riccardi and Kleiner, 1977). Polish studies suggest that most patients fall into the mild or moderate categories, although inherited cases often show more severe symptoms in children (Wojtkiewicz, 2018).

Due to its highly variable expression, even within families, NF1 diagnosis can be complex. However, it is diagnosed when at least two of the following clinical criteria are met: multiple *café-au-lait* macules (CALMs), neurofibromas, characteristic freckles (Crowe’s sign), Lisch nodules in the eyes, specific bone abnormalities, optic glioma, or a first-degree relative with a confirmed NF1 diagnosis (Neurofibromatosis. Conference statement, 1988; Rutkowski et al., 2022). Nearly all individuals develop symptoms over time (Wojtkiewicz, 2018). The original National Institutes of Health diagnostic criteria were substantially revised in 2021 to improve early and accurate detection, introducing genetic testing confirming a pathogenic NF1 variant and updating several clinical markers, including choroidal abnormalities, clarified skeletal changes (e.g., tibial dysplasia), the redefinition of optic gliomas as optic pathway gliomas, and bilateral pigmented lesions (Legius et al., 2021). These updates reflect advances in molecular and clinical understanding and aim to enhance diagnostic precision (Legius et al., 2021; Plotkin et al., 2022).

Although new targeted therapies have shown promise in reducing tumour volume and improving symptom management, treatment for NF1 remains largely symptomatic. The condition is associated with a reduced life expectancy of approximately 8–15 years, primarily due to malignancies and cardiovascular complications (Solares et al., 2021; Na et al., 2024; Staedtke et al., 2024).

Although NF1 is typically diagnosed clinically, and genetic testing is not required in most cases, this clinical pathway relies on physicians’ ability to recognize the characteristic features of the disorder. In Poland, however, many clinicians, including general practitioners and paediatricians, have limited familiarity with NF1, which means that clinical diagnosis is often delayed or missed despite the availability of clear diagnostic criteria (Karwacki, 2020; 2025; Karwacki et al., 2021). This is critical because insufficient clinical awareness directly contributes to the prolonged and complex diagnostic process observed in NF1, with many patients receiving an accurate diagnosis only after several years (Karwacki, 2020; García-Martínez and Hernández-Martín, 2023). The process can also be confusing, emotionally exhausting, and financially burdensome (Ablon, 1999; Kowal and Skrzypek, 2024).

Individuals with NF1 worldwide frequently experience multiple misdiagnoses, the need to consult numerous specialists, and long delays before receiving a definitive diagnosis (Cnossen et al., 1997; García-Martínez and Hernández-Martín, 2023; Dyer et al., 2024). This “diagnostic odyssey” may last for years, contributing to deteriorating health, unnecessary hospitalizations, inappropriate treatments, and substantial emotional strain. At the same time, many individuals and families affected by NF1 internationally face significant systemic barriers in healthcare, particularly in accessing adequately trained professionals, pain management, specialist care, and services aimed at preventing malignant transformation. In Poland, these challenges are further intensified by the limited number of NF1 clinics, especially for adults, substantial travel distances, and a general lack of clinician expertise reported by patients. Access to advanced genetic diagnostics and psychosocial support also remains restricted within the Polish healthcare system (Kowal and Skrzypek, 2024; Karwacki, 2020; Oates et al., 2013; Crawford et al., 2016; Merker et al., 2018; Merker et al., 2025).

This study aims to explore how individuals with NF1 experience the diagnostic process, with particular attention to the early symptoms and how these were interpreted by patients and their families, miscommunications or vague explanations from healthcare professionals, limited access to specialists and genetic testing, in addition to instances of incidental diagnosis, often following the birth of a child with NF1. The specific objectives are to examine: 1) How individuals with NF1 navigated the uncertainty surrounding their diagnosis, 2) How they coped with the diagnostic odyssey, 3) How individuals with NF1 made sense of their condition, and 4) How NF1 influenced their sense of identity.

## Methods

### Study design

While this article draws on a broader research project exploring how individuals with NF1 and their families experience the condition in everyday life, its specific focus is on their diagnostic journey. Given the lack of prior research on this topic, a qualitative approach was adopted. This methodology enabled an in-depth exploration of how individuals with NF1 experience the diagnostic process as well as how it shapes their identity and self-perception. In-depth interviews were conducted to examine how the participants navigate the uncertainty surrounding diagnosis, make sense of their condition, and how these experiences contribute to their process of self-discovery (Supplementary Material S1).

The study description was prepared based on the consolidated criteria for reporting qualitative research (COREQ) (Tong et al., 2007) (see: Supplementary Material S1).

### Ethical issues

The study was carried out in accordance with the guidelines of the Helsinki Declaration of 1964 (updated in 2000) and was approved by the Bioethics Committee of the Nicolaus Copernicus University in Torun, L. Rydygier Medical College in Bydgoszcz (KB resolution 617/2017).

Because interviews were conducted remotely, participants provided verbal informed consent. Before each interview, the interviewer read aloud an information sheet outlining the study’s purpose, procedures, voluntary nature, anonymity, and the right to withdraw at any time. Verbal consent was audio-recorded at the beginning of each interview.

The participants were invited to speak freely, including on sensitive and private matters. They were also informed that they could withdraw from the study at any time or choose not to answer specific questions without any consequences. They were also informed about confidentiality, anonymization procedures, and the legal obligations concerning data protection that were implemented by the researcher and the Data Protection Officer at Jan Długosz University in Częstochowa. Given the potentially emotional nature of some topics, such as past decisions or difficult life experiences, the participants were advised that they could pause the interview if needed. No financial compensation or stipend was offered for participation.

All audio files and transcripts were anonymized by removing personal identifiers, pseudonymizing participants, and modifying or omitting any potentially identifying contextual details. Data were stored on a password-protected, encrypted institutional server accessible only to the main researcher.

### Participants and settings

The study involved individuals with NF1. The following inclusion criteria were used: 1) being over 18 years old, 2) having a medically confirmed diagnosis of NF1, 3) speaking Polish, 4) having access to the Internet, 5) being willing to participate, and 6) providing voluntary informed consent to participate. No formal exclusion criteria were applied beyond not meeting the inclusion conditions (e.g., absence of a confirmed NF1 diagnosis or age below 18).

Recruitment was carried out with the help of two physicians from the Oncology Clinic – Fakomatoz at the Dr Antoni Jurasz University Hospital in Bydgoszcz, who acted as gatekeepers by informing their NF1 patients about the study. Those interested contacted the lead researcher directly and were qualified after confirming their diagnosis.

In total, 93 individuals took part: 69 women (74%) and 24 men (26%), aged between 18 and 64 (M = 36.69, SD = 10.13) (Table 1). The age at diagnosis ranged from infancy to 51 years (M = 17.55, SD = 12.18). No minors participated in the study. Most of the participants were single (58%), while 36% were married. Nearly half (49%) had children, and 65% of their children were also diagnosed with NF1.

**TABLE 1:** Sociodemographic characteristics of participants.

Sociodemographic characteristic	N	(%)
Gender		
Women	69	74.2
Men	24	25.8
Age	Range: 18–64 M = 36.69; SD = 10.13	–
18–19 years	4	4.3
20–29 years	23	24.7
30–39 years	33	35.5
40–49 years	26	28.0
50–59 years	6	6.5
60+ years	1	1.1
Age at Diagnosis	Range: 1–51 M = 17.55; SD = 12.18	–
Diagnosis in childhood (0–12 years)	39	41.9
Diagnosis in adolescence (13–18 years)	17	18.3
Diagnosis in adulthood (19+ years)	37	39.8
Education		
Primary school	6	6.5
Secondary school	45	48.4
Higher education	42	45.2
Marital status		
Single	54	58.1
Married	33	35.5
Divorced	5	5.4
Separated	1	1.1
Place of residence		
A city with over 100,000 inhabitants	41	44.1
A city with up to 50,000 inhabitants	14	15.1
A city with 50-100,000 inhabitants	9	9.7
Village	29	31.2

There were no prior relationships between the researcher and the participants. The researcher had no personal connection to NF1 and approached the study as an independent sociomedical researcher. This outsider position was explicitly communicated to participants before the interviews and was reflected upon throughout the study as part of ongoing reflexivity.

### Research tool

The interview schedule was designed by the first author, who developed a list of potential topics after a thorough review of scientific papers on the experiences of individuals with NF1. Before data collection, the interview guide was reviewed for clarity and relevance. A physician with experience caring for children with neurocutaneous disorders (phacomatoses), including NF1, explored the guide to ensure clinical appropriateness and sensitivity to patients’ experiences.

The categories of questions used in this study included topics related to: 1) early symptoms and participants’ own lay interpretations of these symptoms, 2) inaccurate or non-specific explanations provided by medical professionals, 3) lack of access to specialists and genetic testing, and 4) accidental diagnosis of parents (made after the child’s diagnosis). Additionally, the participants completed several questions on their demographic data (Supplementary Material S2).

### Data collection

The study was carried out between 1 August 2020, and 31 December 2020, by the first author (KK). Due to the COVID-19 restrictions, the interviews were conducted remotely – via phone or online. In total, 95 individuals were recruited and initially agreed to participate; however, two withdrew before scheduling the interview due to a lack of time. Consequently, 93 participants took part in the study. During the initial contact, the participants were informed about the purpose, process, and confidentiality of the study. Only the researcher had access to identifying data, and participation was based on verbal, informed consent recorded during the interview.

To ensure the collection of accurate and meaningful data, the researcher used open-ended, non-directive questions that encouraged the participants to reflect on their personal experiences and the significance they assigned to events related to NF1. Each session lasted around 90 min and was audio-recorded with the participant’s consent. Since the interviews were conducted via phone or online, most respondents participated from their own homes, fostering a comfortable and secure environment for open dialogue.

To ensure anonymity, no personally identifiable data was collected. Each participant was assigned a pseudonym, and any information that could reveal their identity was either removed or modified during the transcription and reporting process. The participants were also reminded not to disclose any identifiable details. All the transcripts and quotations were thoroughly reviewed to avoid any potential indirect identification. These measures were implemented to protect the privacy of both the doctors mentioned in the interviews and the respondents themselves.

A total of 134 h, 26 min, and 35 s of audio material was collected. All audio recordings were transcribed verbatim. In addition to spoken content, the transcripts included relevant paralinguistic elements, such as pauses, hesitations, changes in intonation, or emotional tone, when they were important for preserving the communicative and interpretive context. None of the participants requested transcript verification.

Given the depth and richness of the interviews, as well as the iterative nature of the analytic process, we were able to fully interpret and develop the themes without conducting follow-up interviews. Minor ambiguities that naturally arise in qualitative interviewing were addressed through careful contextual reading, comparison across cases, and analytic memo-writing.

### Data analysis

The data were analysed using Reflexive Thematic Analysis (RTA) following Braun and Clarke’s six-phase framework (Braun and Clarke, 2019; Braun and Clarke, 2021a; Braun and Clarke, 2023; Braun and Clarke, 2024), which offers an interpretive, recursive, and flexible approach to identifying patterns of meaning in qualitative data. The analysis was inductive and semantic, meaning that codes and themes were developed from participants’ explicit accounts rather than imposed through pre-existing categories. The process was grounded in a constructivist-interpretivist paradigm, aligning with the qualitative descriptive orientation of the study.

Analysis began with familiarisation, during which the first author repeatedly read the transcripts and made preliminary analytic notes. Open, descriptive codes were then generated manually, capturing meaningful features of participants’ accounts related to the diagnostic experience. These codes were developed into candidate themes through an iterative and reflective process that involved grouping them by conceptual resonance rather than following predefined categories. Themes were reviewed, refined, and reworked recursively through close rereading of the data, ensuring their internal coherence and clear distinction from one another. Illustrative extracts were selected only after the themes had been analytically defined, to illuminate rather than determine their meaning.

All coding was conducted manually, without the use of qualitative analysis software, which is consistent with the close, interpretive engagement emphasised in RTA. In line with RTA, themes were not determined through saturation-based criteria or frequency counts. Consistent with the principles of RTA, themes were also not examined according to demographic subgroups, and no proportions of respondents were used to determine their relevance; RTA focuses on patterns of meaning rather than quantifying or comparing participant categories. Instead, they were developed through iterative, recursive engagement with the dataset until they demonstrated analytic sufficiency, internal coherence, and clear conceptual distinctiveness (Braun and Clarke, 2021b; Braun and Clarke, 2023). This reflexive process prioritised depth of meaning and interpretive clarity rather than the notion of “data saturation,” which Braun and Clarke explicitly caution against in RTA.

Although the data were collected and coded by the first author, the second author was actively involved in reviewing and consulting on the analysis to ensure the credibility and rigor of the study. Both team members contributed to maintaining data-grounded interpretations, thereby minimizing potential bias. The researchers also engaged in ongoing reflexivity throughout the analytic process, using memo-writing and critical self-reflection to remain aware of how their interpretive position might influence coding and theme development.

## Results

The analysis generated eight key themes that capture the complexity of the diagnostic experience of individuals with NF1 (Table 2). These themes include: 1) the early symptoms and lay interpretations; 2) non-medical explanations provided by clinicians; 3) parental responses and uncertainty during the diagnostic process; 4) active pursuit of diagnosis by the patient and their family; 5) diagnosis (un)written into the family; 6) accidental detection of a parent’s disease during the child’s diagnosis; 7) from phenotypic diagnosis to genetic testing; 8) (un)expected and (un)wanted diagnosis.

**TABLE 2:** Themes developed through reflexive thematic analysis.

Theme	Brief description
The early symptoms and lay interpretations	How visible signs such as CALMs and lumps were normalized within families and interpreted through everyday, non-medical frameworks
Non-medical explanations provided by clinicians	How vague, non-specific, or everyday explanations from physicians contributed to uncertainty and diagnostic delay
Parental responses and uncertainty during the diagnostic process	How parents’ hesitation, limited awareness, emotional strain, and lack of guidance shaped early diagnostic experiences
Active pursuit of diagnosis by the patient and their family	How patients or family members took on diagnostic agency, navigating fragmented care and seeking specialist input
Diagnosis (un)written into the family	How familial inheritance, intergenerational narratives, and the presence or absence of family history shaped the recognition and meaning of NF1
Accidental detection of a parent’s disease during the child’s diagnosis	How parental diagnosis occurred unexpectedly during a child’s evaluation
From phenotypic diagnosis to genetic testing	How participants experienced transitions from clinical to molecular testing, and how genetic confirmation shaped identity
(Un)expected and (un)wanted diagnosis	How diagnosis emerged suddenly (e.g., after acute symptoms or accidents) and the emotional reactions accompanying it

### The early symptoms and lay interpretations

The first symptom of NF1 – *café-au-lait macules* (CALMs) – does not usually trigger efforts to obtain a diagnosis. When this symptom appears as a single mark, it is often normalized by individuals with NF1, their parents, and family members (i.e., treated as ordinary and insignificant). It is usually noticed by the mother, who is the first to naturalize these symptoms (i.e., interpret them as innate or “one’s nature”). The naturalization of symptoms is reinforced by the presence of similar skin changes in the parents or grandparents of the individual, which leads to CALMs being perceived as a hereditary feature of appearance rather than a potential symptom of a disease.

The lack of awareness of the disease, or of health-related issues more generally, often shaped how individuals with NF1 and those around them interpreted early symptoms, which were frequently perceived as non-urgent. This results in normalization and domestication of the disease. Single CALMs do not raise concern as they cause neither pain nor other discomfort. They are interpreted as something as common as freckles or birthmarks. During childhood and adolescence, the presence of these symptoms has no significance for experiencing one’s own body. On the contrary, they become inscribed into the individual’s “natural” bodily identity, rather than into the framework of illness.

*I never worried about the spots. I even have one on my cheek. Everyone has some spots.* (Interview 18)

*It was during my teenage years, when I was 13–14 at the time. I was in primary school. The first changes that had always been with me, which I never associated with any illness, were the café-au-lait spots. They didn’t evoke any emotions in me.* (Interview 25)

In the pre-diagnostic phase of disease awareness, lay interpretations emerge regarding the causes of the spots on the participants’ skin, which serve to normalize the symptoms and do not trigger diagnostic actions. In these accounts, parents, like the participants themselves, did not recognise the spots as a possible medical sign, drawing instead on familiar, everyday explanations.

*I asked why I had those little bumps or spots, and my mother said that I was simply born that way, that she had had them since childhood, and that her father had them too, so that’s just how it is (…). She kept telling me that she was born that way, her father had it, so I must have inherited it from them. She never said it could be a symptom of some illness or anything like that. Later, I also got used to the idea that this is simply who I am and what I look like.* (Interview 44)

*I had café-au-lait spots on my body. (…) In my hometown, where I was born, in* [name of town]*, back then, they used to wash children with water poured from the kettle, and my family claimed that I hadn’t been washed properly, and that’s why I had such spots on my body. And secondly, in the place where I lived, there were superstitions that if a pregnant woman got scared of something, or, for example, walked under fence wires in a pasture, such spots would also appear on the child.* (Interview 5)

The second symptom of NF1, which the participants perceive as a normal feature of appearance rather than a disease, is subcutaneous neurofibromas. The “lumps”, as the participants call them, are not associated with a serious illness either.

*Until the age of 39, I didn’t know it was NF1. Absolutely nothing. The spots were there, the lumps were there. Nothing happened at all.* (Interview 20)

*There were lumps around the chin and jaw area, like little beads, something like that, under the skin. But it wasn’t anything worrying. No one even noticed it. No one paid any particular attention to it or considered it might be the beginning of some illness.* (Interview 76)

Although subcutaneous neurofibromas become an increasingly troublesome element of one’s “appearance” as a person grows older, they are still interpreted as a cosmetic defect unrelated to disease. When they reach a large size, they become aesthetically distressing. The participants express a desire to have the skin lesions removed, though the motivation remains aesthetic rather than related to any perceived physical health risk. Both participants and the clinicians they consulted typically regarded these “lumps” as harmless cosmetic issues, and thus, they were surgically removed without raising any medical suspicion or concern.

*I happened to grow some lumps. I had a lump grow on my forehead that looked almost like a horn; it looked terrible, so I finally decided to have it removed. It kept growing. (…) It was more about aesthetics that made me decide to remove it.* (Interview 6)

*When I was a girl, I had quite a large lesion on my labia. And I still didn’t know that it was related to the disease. I just went and had that lump-like lesion removed.* (Interview 32)

### Non-medical explanations provided by clinicians

CALMs were also downplayed by doctors. The participants reported that they often heard comments like “that’s just your nature” or “the spots are nothing serious.” The fact that, despite visible clinical symptoms, a diagnosis was not made for a long time reflects what participants perceived as gaps in clinicians’ familiarity with NF1 and the challenges of recognizing the condition in routine practice. The spots were defined as harmless pigmentary defects, “birthmarks,” or “natural features of appearance”. Doctors reassured the participants that the issue did not require medical intervention as it would resolve on its own.

*After I turned four years old, I started developing birthmarks. But when my mom asked the doctor about it, the doctor just said, “That’s your nature, just your nature,” plain and simple. Another doctor simply asked, “What is that?” And yet another doctor, when my mom mentioned these spots had appeared, just said, “Ah,” and nothing more came of it.* (Interview 7)

*From what I remember, my mom said that when she asked the doctors why I had these spots, they said it was simply because when she was pregnant, the pigment didn’t spread properly, and that’s why I have these spots. The disease was diagnosed about 8 years ago, which means I was around 26 years old.* (Interview 48)

*Since I was a child, I have had such spots on my skin. The family doctor told my mom that they were just birthmarks and there was nothing to worry about. And so I lived carefree for 13 years with that information.* (Interview 24)

According to the participants, the gaps in clinical knowledge and limited familiarity with NF1 mainly concern doctors working in small, local primary care clinics. Many of the respondents emphasized that primary care physicians were not prepared to recognize and manage patients with NF1, which resulted not only in missed or incorrect diagnoses but also in a reversal of roles in the doctor-patient relationship. Patients and their families became the “experts by experience,” forced to independently seek information and educate the specialists.

*Of course, I had those spots first, but the first doctor, the primary care physician, didn’t know much about it. He didn’t explain what it was because very few doctors knew what it actually was. The primary care doctor didn’t pay much attention to it. (…) He saw the spots but said nothing about them. “Well, that’s how it is,” “they’re just like “little mice”, “people sometimes have things like that.”* (Interview 23)

*My parents basically taught themselves about this disease from encyclopaedias and libraries. My parents ended up teaching the pediatricians here what kind of disease it is. They would nod, as if to say they knew and didn’t need to be told anything, but in reality, they knew nothing. (…) Even now, there is still a big problem with doctors’ knowledge about our disease here* (Interview 9)

When discussing the lack of substantive preparation among medical staff, the respondents also pointed to generational differences among doctors. Younger doctors appeared better prepared and more familiar with NF1, while some older clinicians were perceived by participants as having more limited or outdated knowledge and needing to update information during the consultation.

*When I went to see an ophthalmologist in* [city name] *and told her that I have Lisch nodules, because I knew I had them, she said, “Please step outside for a moment.” She called me back shortly after. There was a book open to the section on Lisch nodules – she had to read up on what they were. I’ll say this: older doctors don’t really know what it is. But younger doctors, as soon as I walk in, they immediately know, they have experience with these patients and understand what the disease is.* (Interview 33)

According to participants, skin symptoms were sometimes not fully recognised as indicative of NF1 and were initially interpreted as signs of other conditions, for which treatment was believed to be necessary to address the persistent spots.

*I think the doctors were not prepared to take care of me. When I said that something new had appeared, and it was a neurofibroma, the doctor said, “Please don’t worry, because it appears and sometimes goes away on its own.” The doctors at the hospital in* [city name] *also said that nothing needed to be done about it.* (Interview 7)

*The doctors claimed that the CALMs might be related to the kidneys. They said that if the kidneys were treated, the spots would disappear. Never, ever during my childhood was I diagnosed with the possibility that it could be this disease. I found out when I gave birth to my second daughter, at the age of 23.* (Interview 49)

According to some participants’ accounts, the observed CALMs did not prompt appropriate diagnostic initiatives from medical staff, which participants described as contributing to feelings of uncertainty and disappointment regarding their diagnostic experiences. Participants perceived that some clinicians used non-medical or everyday explanations that contributed to the normalization of the spots, as evidenced, on one hand, by their lack of questions and inquiries, and on the other, by excluding the possibility of the disease and misinterpreting subcutaneous neurofibromas as acne.

*When I was in the hospital with my son in the pregnancy ward, before delivery, the doctors, while doing an abdominal ultrasound, asked, “Why do you have such a colourful belly?” And I said, “I don’t know, I was just born like this.” And that was it. No one dug deeper or asked any more questions. (…) Not one of them even suggested that something might be wrong. So I thought it was just my nature.* (Interview 44)

*I had these nodules on my body. At first, they were just small dots. They had been there earlier, but were visible. But it was always considered teenage acne. But I was already older, so it probably couldn’t have been acne, yet every dermatologist always said, “Impossible, it can’t be that disease, it’s acne.” And wherever I went to a dermatologist, they claimed it was impossible that I could have something like that.* (Interview 11)

When attempting to determine the causes of reported symptoms, participants felt that some explanations resembled everyday or non-medical interpretations and did not prompt further diagnostic action. Instead of initiating thorough diagnostics, they relied on common-sense justifications that often resembled stereotypes and beliefs existing outside of medicine. Skin changes were explained by sweat, water, or sun exposure, and their presence was treated as natural or incidental phenomena that did not require further consultation. Such interpretive strategies not only delayed the moment of making an accurate diagnosis but also lowered diagnostic vigilance and reinforced the belief in the harmlessness of the symptoms, both among the patients and their families.

*I didn’t know at all what was happening. I had these little spots. The doctors didn’t know what it was. They had no idea. They thought it might be from the water, maybe sweat. They themselves didn’t know whether it was a genetic disease or something else.* (Interview 31)

*My mom recalled that she had taken me to various places, and no one was really able to determine that it was NF1. There were some suspicions. Doctors said the spots were from the sun, so everything dragged on like that.* (Interview 38)

On the other hand, even when specialist doctors, including pediatricians or surgeons, indicated the need to remove subcutaneous neurofibromas, this was often due to their mistaken identification of these tumours as lipomas or sebaceous cysts. They were not associated with neurofibromatosis in any way and did not trigger further diagnostic procedures.

*I had tumours; I had a neurofibroma removed when I was 18. (…) The doctor said it was a lipoma. The moment he saw me, he said it was a lipoma and that it would be removed. No doctor connected it to anything else. There wasn’t even any thought that it could be a disease. They simply found a neurofibroma on my head and removed it. That was it.* (Interview 93)

*I remember that when the first changes appeared, I went to a doctor, a paediatrician, who then referred me to a surgeon at the local health centre. The surgeon assessed the lump not as a neurofibroma but completely as a sebaceous cyst.* (Interview 25)

Spontaneous limb fractures without mechanical injury were also a medically misunderstood symptom of NF1. They mark the beginning of a diagnostic journey marked by many misdiagnoses and incorrect explanations. The failure of bone healing often remained medically unexplained from the patients’ perspective for a long time. Other bone symptoms, including back pain, are not associated with neurofibromatosis either, but are instead attributed, for example, to prematurity.

*When I was six months old, my mom, while lifting me from the bed, heard a snap of the bone. And it was very strange because my leg just broke, as if by itself. (…) The cause was unknown. The doctors said, “Ma’am, we don’t know what’s going on. The leg isn’t healing, there may be necrosis, and we might have to amputate.” My mom said they were irresponsible and took me out of the hospital.* (Interview 63)

*Because I was generally a premature baby. From the age of 12, I had back pain. The doctors blamed it on me being very small as a child, on developmental delay. They attributed everything to prematurity. I was only diagnosed with NF1 when I was 20 years old.* (Interview 27)

Like CALMs, fractures, and bone pain also appear as “invisible” symptoms. Nonetheless, if properly interpreted, they could have triggered a diagnosis. The above accounts from the participants demonstrate the existence of a “diagnostic gap”. The presence of bone symptoms is not, for doctors, a sufficient reason to initiate diagnostic procedures.

Lack of proper diagnosis in the early stages of the disease resulted in doctors attempting to treat the symptoms reported by the patient. According to participants, symptomatic treatments recommended by specialists, including surgical interventions later perceived as unnecessary, did not lead to health improvement and, in some cases, were reported to coincide with deterioration or episodes of severe health crises.

*That was my biggest spot. My mom took me to a dermatologist. He told me to use some ointment, some kind of jelly for it. However, he did not diagnose the disease. I remember the dermatologist gave it to me.* (Interview 30)

*When I was 20, I went to a gynaecologist because my breasts hurt. He did an ultrasound and gave me hormonal medication to reduce the pain. I took it for six months, but it didn’t help, so I got a second medication. I felt very, very bad because I gained two dress sizes in two days. So I went back to him to tell him what was happening. The doctor said I should stop taking the medication, but since the drugs weren’t working, he said he would have to surgically remove my ovary. I didn’t agree. (…) After a long search for a diagnosis among gynaecologists, oncologists, and surgeons, I finally saw a neurologist who identified it as neurofibromatosis type 1 and told me I needed to be under the care of a diagnostic clinic.* (Interview 27)

*It was epilepsy, but the doctor thought it was febrile seizures. I travelled to* [name of big city]*, where there was a doctor who treated me for epilepsy. In* [name of another city]*, I came under the care of a good specialist, a doctor who treated me with medication, but it wasn’t effective. (…) The result was that I was close to dying.* (Interview 67)

Participants perceived that limited familiarity with NF1 sometimes contributed to interactions they experienced as stigmatizing, which translated into feelings of isolation and harm. Such reactions, as perceived by participants, stemmed from limited awareness, misunderstandings, or stigma, and resulted in feelings of humiliation, loss of dignity, and undermined the participants’ trust in the healthcare system.

*One doctor started off by saying that I could play roles like that in movies, that people might be afraid of me. That really shook me, so to speak. (…) Later, in adulthood, after a car accident, a nurse said she had to wear two pairs of gloves so she wouldn’t catch anything from me. The doctors – an endocrinologist and an orthopaedist – diagnosed a skin infection and said that once I’m cured, I should come back. It was such a shock to me that a doctor would say things like that.* (Interview 33)

The skin and bone symptoms described above did not lead doctors to take further diagnostic actions or to refer patients to appropriate specialists. The information provided to patients about changes in their bodies was brief, and the treatments undertaken were ineffective. Despite numerous medical consultations, the diagnosis of NF1 was made relatively late.

### Parental responses and uncertainty during the diagnostic process

In isolated accounts, some parents initially perceived visible symptoms as benign or cosmetic, reflecting limited awareness of NF1 and the absence of publicly available information at the time. Owing to what participants described as limited recognition of NF1-related symptoms and variable levels of clinical awareness, as well as reassuring, vague, and sometimes contradictory clinical explanations, parents in some cases paused or slowed their search for an accurate diagnosis. According to participants’ accounts, these decisions were often shaped by limited guidance, mixed medical messages, or the absence of publicly available information about NF1. In such situations, families frequently relied on their own or clinicians’ lay interpretations of early symptoms. This combination of unclear information and a lack of consistent diagnostic direction meant that even when parents sought answers, they often did not receive clear explanations, which contributed to discouragement. For some individuals, this resulted in long gaps, sometimes extending into adulthood, before they re-entered the diagnostic pathway and received a confirmed NF1 diagnosis.

*Since it was just “my nature”, I didn’t pay much attention to it. Later, it started to worsen, and I began to grow up and became more interested in it myself. But, as I said, I saw several doctors, and none of them showed any interest or gave any guidance, so I gave up on it, too. When ten doctors told me, “That’s just your nature,” I decided to leave it alone. (…) That’s why I only found out about the diagnosis when I was 27 years old.* (Interview 36)

*My parents went from doctor to doctor, but they didn’t know what was wrong with me. Later, my parents gave up because if the doctors didn’t know, then it was probably just some cosmetic defect.* (Interview 12)

For the respondents, the early stage of life with the disease was marked by uncertainty and a lack of knowledge. On one hand, there was a lack of a coordinated or systematic approach to early symptoms, including limited access to specialised diagnostics, which contributed to prolonged uncertainty and worsening health problems. On the other hand, the interviews revealed that parental responses were sometimes hesitant or fragmented, reflecting limited awareness of NF1 and uncertainty about the significance of emerging symptoms. As a result, children often navigated ambiguous bodily changes without consistent guidance or clarity. Together, these dynamics contributed to a delayed diagnosis.

*The first time I went to the hospital was when I was eleven because I had something above my eye. I have dark eyes, sort of bluish, you could say. There was a birthmark, like coffee with milk, and it started growing thick. So I ended up in* [city name]*, at the hospital, and they took a biopsy, but they didn’t say what it was until several years later. Because my mom and dad kind of neglected it. And at the age of twelve, it was diagnosed as some kind of mark, but they didn’t know what it was and just left it like that. But it started growing from the age of twelve.* (Interview 10)

*My parents didn’t do any tests for me; I didn’t know if I had a confirmed NF1 diagnosis or not. Only later. At first, I had very few nodules, but now there are so many.* (Interview 34)

In such accounts, participants perceived parental inaction as a lack of sufficient response, although these experiences occurred in a context of limited information, absence of guidelines, and restricted access to specialists at the time. What participants interpreted as parental passivity, despite the child’s deteriorating health, often had deeper psychosocial underpinnings. One of these was the silence or taboo surrounding illness within the family, particularly when NF1 was hereditary and present in other relatives.

To illustrate that these perceptions were not limited to a single case, but appeared across several narratives, we also include the following participant accounts:

*I received my diagnosis at the age of 34. (…) Well, it was a bit of a shock. This was actually my parents’ fault, because they simply neglected it. Because they knew about this disease. They knew about this disease. I told my mother. (…) When I asked her whether she knew anything about it, she said she did. And I had all the symptoms. More and more spots every year, learning difficulties, and some small lumps appearing on my hand.* (Interview 93)

*I definitely inherited this disease from my father. (…) My dad definitely had NF1, so my mother noticed it in me right away, because there were these small lumps. The disease was most aggressive during puberty. My mother didn’t do anything about it; she didn’t support me in it. On my own, as a young girl, I wanted to look for doctors. (…) I didn’t have that kind of support. My family situation was such that my mother didn’t really want to deal with it. She said that since I was going to school, I had arms, I had legs, and then everything was fine.* (Interview 73)

In these situations, delayed diagnosis stemmed from an interplay of systemic and familial factors, including the fact that parents or caregivers sometimes struggled to recognise or interpret progressing symptoms. Participants also recalled situations in which they were not fully included in the diagnostic process, receiving limited information about tests or medical decisions. Such a paternalistic family communication style reduced transparency and constrained the autonomy of individuals with NF1.

The mother frequently appeared in participants’ narratives as the primary caregiver and main decision-maker regarding health-related actions. In some cases, mothers acted as initiators of the diagnostic process, while in others they served as mediators of information, deciding when and how to communicate health-related issues. These patterns partly reflect broader caregiving expectations within Polish families, although roles varied substantially across households.

*The doctor issued referrals, but my mother kept delaying. I suspect she probably had an idea of what was wrong with me, because my father was also ill with it. My father died at the age of 35 due to complications, as he had a tumour in the mediastinum. (…) After a biopsy of my tumour, there was a diagnosis of what it was, but that result disappeared entirely. I never saw it. I remember it was written in Latin, but later I could no longer find it. Apparently, it simply vanished because my mother must have hidden it. My mother didn’t want to let me go to the hospital at all, saying that people die in hospitals. (…) I didn’t know what was wrong with me, and I especially resented my mother for doing nothing about it. I felt there was no one to help me, because I had no one to turn to – my father had died when I was not yet five years old. (…) I knew something was wrong, and I was afraid it might be cancer, just like my father had.* (Interview 28)

Mothers’ hesitation to seek or disclose the NF1 diagnosis can be understood as reflecting emotional strain and difficulty in confronting potentially distressing information, particularly given concerns about hereditary transmission. In one of the accounts, even after the diagnosis was formally established, the mother assumed a protective, gatekeeping role and chose not to disclose it immediately. Such patterns of silence within families, especially when hereditary illnesses are involved, may create ambiguity, emotional tension, and a sense of isolation for those affected.

### Active pursuit of diagnosis by the patient and their family

Misinterpretations of symptoms by doctors – whether incorrect, non-medical, normalizing, or dismissive – often result in the patient or their relatives taking on the responsibility of securing a diagnosis themselves. In many cases, an NF1 diagnosis is the outcome of the personal determination of the parents, caregivers, or the patient (often in adulthood), rather than the result of an organized healthcare system process.

*As a child, I already had the first symptoms and was being tested for this disease around the age of 10 or 11, but at that time, the disease was ruled out in my case. (…) Later, I was diagnosed because I had a difference in leg length. There was a change on my leg that the doctor described as lymphatic edema. And later, actually in adulthood, I was looking for some way to get rid of this lymphatic edema. I ended up with a doctor who referred me for genetic testing to diagnose me for NF1. And it turned out that it was neurofibromatosis. I was 29 years old then.* (Interview 39)

*I went to various doctors within the National Health Fund, but they said that since I’m alive, there’s nothing wrong with me. And then bigger problems with my spine started, my bones and joints hurt. Eventually, I had the opportunity to sign up for private healthcare at* [name of private healthcare network]*. I started seeing doctors and came across a dermatologist who diagnosed me right away. And since then, it has been a cascade of doctors, tests, and consultations.* (Interview 36)

Diagnostic chaos, the lack of clear guidance from primary care physicians, and limited access to specialists mean that patients and their families must obtain information on their own, initiate genetic testing despite the high costs, and navigate the private healthcare system. Faced with institutional helplessness and the absence of reliable medical information, they are the ones who make decisions about further consultations, seek out specialists, and look for therapies that could address the constantly changing symptoms. Thus, the final, formal diagnosis of NF1 appears not as the result of cooperation with the system, but as the outcome of an individual’s struggle to understand one’s own health situation.

*The neurologist said it is a very rare genetic disease, but they themselves knew very little about it. The doctors told me to look for information about this disease on my own. (…) I said to myself, it’s about time to find a hospital in Poland where they treat this disease. I wrote emails to all the hospitals in Poland. Every hospital got an email. Only the hospital in* [city name] *responded. And I was immediately scheduled for an appointment there.* (Interview 5)

*I wanted to help myself. I was looking for information on whether I could be helped. I was looking for doctors, but no one was able to help me. (…) I started having very severe back pain, so I was looking for a doctor who could prescribe me a different medication because they understood this better.* (Interview 27)

Participants reported that the search for a diagnosis was often triggered by increasingly noticeable or alarming symptoms, including rapidly growing tumours around the eye sockets or oral cavity, vision impairments, drooping eyelids, brittle bones, and motor difficulties. Because of their significant impact on daily functioning, the parents decided to seek consultation at a specialized centre where the diagnosis of NF1 was made, although this sometimes occurred under accidental circumstances. The presence of CALMs (café-au-lait macules) then served as confirmation of the diagnosis.

*My mom noticed that my left eye was protruding. The first symptom was that I started walking strangely. When my mom went to the eye doctor, at first, they only wanted to prescribe glasses. But when my mom said that I had these spots and she didn’t know what they were, at that moment, they stopped and referred me to* [city name]*, where an MRI was done. They detected an optic nerve glioma in my left eye. I was three and a half years old then.* (Interview 46)

*On the right side, I had swelling near my salivary gland, down below. I went to the dentist. It turned out it wasn’t related to a tooth. They sent me for an ultrasound. And on the ultrasound, they found a lump, some growth, and I was told I would need surgery. (…) They removed that tumour. And after the histopathological examination, they said it was NF1.* (Interview 24)

Once again, the mother plays a key role in seeking information about a potential illness. As the guardian of health and caretaker of the family, the mother emerges as a central figure in the process of obtaining medical assistance and reaching a diagnosis – demonstrating determination, agency, and a critical attitude toward the healthcare system. In many cases, it is precisely the mother’s persistence that becomes the driving force behind the entire diagnostic and therapeutic process.

*My mom searched for help for me and my leg for a long time. I was in a constant state where my leg was broken. She kept searching for a long time. (…) She found something in* [city name] *— she happened to read in a newspaper about some innovative method of stimulation with electrical currents. And it helped. The fact is, it took quite a long time because I was in the hospital for about four years. (…) Later, when I was already in primary school, my mom noticed that I was much shorter than the other children. So my mom arranged some appointments with doctors in* [city name]*, with geneticists. The geneticist examined me, noticed the café-au-lait spots, asked me if I had had problems with my bones, and so on. They measured my calcium levels, checked for nodules on the fundus of the eye, and started doing MRI scans. And that’s how it turned out that I was diagnosed with NF1.* (Interview 63)

*My father was practically not involved in my illness. He didn’t travel with me to the doctors as often as my mom did since I was a child. My mom has been much more present — present in my illnesses and in my whole story. At the age of 15, I had high blood pressure, and during every school break, I would go to the nurse to have it measured. And back then, no one thought of doing an abdominal ultrasound. My mom was the one who arranged that later. The ultrasound revealed tumours, but the doctor didn’t interpret it correctly. It was my mom who figured out that the doctor had misread it.* (Interview 90)

For an adult living with NF1, the motivation to seek medical help often comes from bodily changes that become visually distressing, start to cause pain, or limit daily functioning. This primarily refers to disfiguring tumours on the face, chest, buttocks, or limbs. They are accompanied by a growing sense of discomfort and difficulties in accepting one’s appearance. The body altered by NF1 becomes a burden — a source of shame and frustration — forcing individuals to cover up and conceal embarrassing defects to reduce social exposure.

*Back then, as a young woman, I was embarrassed because I couldn’t wear anything I wanted – my calf was deformed, so I didn’t wear short skirts. Later, the largest spots, similar to the one on my calf, appeared on my collarbone – both on the left and the right side. These spots on my collarbones deepened and became raised. They caused me so much pain that, sometime before I turned fifty, I could no longer wear anything with a neckline. I had to wear a scarf, because if I wore something with a deeper neckline, these changes would be visible. Later, these changes also caused me severe pain. They felt like jelly and were protruding, so they also pressed and rubbed (…). So I went to see him* [the doctor] *and found out that it was Recklinghausen’s disease, or NF1. I was exactly 50 years old then.* (Interview 30)

Taking personal responsibility for one’s health was, for some participants, a reaction to earlier difficulties within the family in recognising or addressing emerging symptoms. For many respondents, entering adulthood meant the need to independently arrange medical care and acquire knowledge about the disease. The final diagnosis of NF1 came late, which undoubtedly influenced the way the illness was experienced and understood. For those who, in childhood, did not receive sufficient help or involvement from caregivers in the diagnostic process, the diagnosis of NF1 was the result of their own determination.

*I started to realize on my own that I should be looking for some kind of help, getting some answers to my questions, because my parents kind of neglected the matter. I don’t hold a grudge against them – it’s just that my parents had me very late in life. (…) And I think they simply didn’t have the strength to take me places. So I was left to deal with it on my own. I started taking an interest in it myself, I began looking for a neurologist on my own, and I started asking for referrals myself.* (Interview 17)

*In fact, I only began trying to get treatment after turning eighteen. When I turned eighteen, I started going to doctors on my own – mostly to ophthalmologists, to find out what it was and how to get rid of it. Because, practically speaking, my mom and dad had somewhat neglected it. At the age of twelve, I was told it was some kind of birthmark and that the doctors didn’t know what it was. And it was left like that, but from the age of twelve, it started to grow.* (Interview 10)

### Diagnosis (un)written into the family

The inheritance of the disease within a family often serves as the starting point for a presumed diagnosis. In this context, genetic burden becomes a diagnostic aid–based on observed symptoms, the respondents are aware of the illness even before receiving a formal diagnosis. Moreover, they are conscious of its hereditary nature. In such cases, the identification of NF1 is based on the family’s medical history, and knowledge about the disease is often passed down by older family members or drawn from observations of their bodies. The respondents recognize similar traits and symptoms in themselves, intuitively diagnosing the condition, even if no medical diagnosis was made in the previous generation. This illustrates the crucial role of the family context and the beliefs transmitted through intergenerational relationships in the diagnostic process. Awareness of the disease’s presence in the family can significantly accelerate diagnosis and foster the patient’s self-identification, even before a medical confirmation is obtained.

*I didn’t need a diagnosis because I knew I had this disease. Before it was formally diagnosed, I already knew what the disease was called. My dad had this disease. I inherited it from my dad.* (Interview 4)

*I always had these eruptions on my body during adolescence – little lumps. My mom had this disease, and I figured I had inherited it, because at that time, my parents hadn’t done any tests. It wasn’t confirmed. I’d had it since birth. In our family, my mom and my brother were affected.* (Interview 34)

What can delay the medical confirmation of the disease is the misinterpretation of symptoms in members of the previous generation. Skin changes or lumps are not attributed to a disease, and, as a result, no attempts are made to treat them. Some respondents denied having a family history of NF1, yet at the same time mentioned that their relatives had “sebaceous cysts” or “lipomas.” Similarly, even frequent cases of cancer within the family did not raise diagnostic awareness. These accounts point to a lack of knowledge about the specific nature of NF1 and an absence of awareness of its possible hereditary character.

*My mom also had lots of lumps, but back then, I don’t think anyone really did anything about them. My mom never got treated for it. She also died early of breast cancer at the age of 45. (…) I got my diagnosis when I was 34, after having a tumour removed from my leg.* (*Interview* 88)

*No, no one else in the family had it, ma’am. If they had anything, it was lipomas. Everyone had either a sebaceous cyst or a lipoma. But to have what I have – no. In our family, there are just a lot of cancer cases. (…) I only found out about my diagnosis at 44, when my son was born.* (Interview 31)

Although inheriting the disease from a parent can simplify the diagnostic path for NF1, it also triggers strong emotions, primarily feelings of grief toward the “genetic culprit.” There is blaming of the parent, a sense of injustice, and even feelings of being “stained” or “tainted” by the inherited disease. The respondents who experienced this often compared themselves to healthy siblings, questioning the randomness of inheritance. The transgenerational nature of NF1 also raised fears that the disease would be passed on to their own children.

*I felt some resentment toward my father for passing it on to me, like he stained me or gave me some flaw. I was simply angry at him for burdening me with this disease instead of my brother or sister. When I had those lumps, I had big complaints toward him, wondering why it had to be me, why not my older sister or younger siblings – why did he have to pass it on to me? And now I’m afraid that I’ll pass it on to my child in their genes.* (Interview 85)

In trying to understand the inheritance of NF1, the mother – being the more active party in the diagnostic process – draws on non-scientific beliefs, formulating family speculations about the causes of the disease. Such an attempt to understand and explain its origin reflects the need to rationalize and make sense of the unexpected presence of the disease in one of the children. In one narrative, an anecdotal hypothesis appears: the respondent’s mother linked the absence of the disease in the other children to the consumption of alcohol at the time of their conception, as opposed to the “sober” conception of the child with NF1.

*I inherited the disease from my dad. But I have it, and my sister and brother don’t. Maybe alcohol is what kills the disease. Because my brother and sister, according to what my mom said, were conceived after a party, after which my mom got pregnant. In my case, though, the conception happened sober. I haven’t consulted anyone about this, but I think there might be something to it – after all, alcohol kills a lot of different things.* (Interview 4)

An early diagnosis of NF1 can be challenging when the disease is not hereditary. In cases where NF1 results from a spontaneous gene mutation, the affected individuals often experience feelings of isolation. The absence of a family history of the disease triggers a sense of “initiating the disease” within them. Above all, the lack of a familial background prevents earlier recognition, as there are no coping patterns or preparation for the diagnosis. The disease becomes part of an identity that cannot be anchored in any known family narrative. For many respondents, this was associated with feelings of alienation, loneliness in the diagnosis, and an awareness of their own “genetic uniqueness.”

*No, no one in my family was ill. I’m the only one in the whole family. It just happened to be me. Someone has to be the first.* (Interview 9)

*Nothing in the interview indicates anyone was sick in the family. It looks like I’m the first case.* (Interview 52)

*No, no one in my family is sick. I started this “wonderful procession.”* (Interview 42)

### Accidental detection of a parent’s disease during the child’s diagnosis

The lack of a medically confirmed diagnosis of NF1 in a parent as a hereditary disease sometimes leads to situations where neurofibromatosis is only recognized at the time a child is diagnosed. In such cases, the child becomes a kind of “mirror” reflecting the parent’s health status and serves as the basis for making the parent’s diagnosis. Interest in their own medical history arises only after a suggestion from the paediatrician, rather than as a result of earlier attempts to identify the causes of changes in their own body and diagnostic efforts. Therefore, the parent’s diagnosis is accidental – it is the recognition of the child’s disease that acts as the diagnostic trigger, prompting the parent to examine their own body and take further medical steps.

*I never got diagnosed myself; it just happened that when the kids were being diagnosed, the doctor found out that I also have Recklinghausen’s disease.* (Interview 3)

*Actually, I found out about the disease only three years ago through my daughter, who inherited it from me. It started when I was at the doctor’s appointment with my daughter. The doctor asked what those spots on her body were. I said she got the birthmark from me. The next time I went to that doctor, she told me to check if maybe I have that disease too. I looked through my medical history and realized I had had congenital pseudarthrosis since childhood, which is something that can happen with this disease.* (Interview 93)

The diagnosis of NF1 is therefore often an indirect and surprising experience–some respondents, despite having earlier symptoms, only learn about the disease in adulthood after it has been diagnosed in their own child. In such cases, awareness of their own condition emerges secondarily to concerns about their child’s health. Although the reaction to such a revealed genetic diagnosis often involves shifting the focus of the disease from the parent to the child (with the parent concentrating on the child’s illness rather than their own health), strong emotions also accompany this process – surprise, grief, and feelings of being insufficiently supported by medical professionals. These emotions stem from the lack of diagnosis in childhood, awareness of misinterpretations of symptoms by doctors, and the general lack of medical knowledge about NF1. The respondents emphasized that the lack of knowledge about the disease and its hereditary nature influenced their reproductive decisions. The absence of an earlier diagnosis prevented emotional and practical preparation for the possibility of passing the disease on to offspring.

*I found out I have the disease when I gave birth to my second child. Before that, I was never diagnosed with it. I had some spots on my body, and I’m quite short – only 152 cm – but the doctors said some people are just small and there’s nothing wrong. So when I had my second child, I pushed to get myself examined and diagnosed because my younger daughter was born with a large tumour on her face. And from that moment, the fight to get diagnosed began. When my daughter was 9 months old, the diagnosis came: all three of us have NF1. (…) When you know what you’re dealing with, you can sort things out in your head much better. Otherwise, it just hits you – “boom”. And then you’re responsible not only for your own health but also for your children’s. When you know you’re sick, you make conscious decisions. As for the doctors who didn’t diagnose me earlier, I hold a grudge.* (Interview 49)

*I don’t know why no doctor has ever diagnosed this disease! People go for regular check-ups at work, visit doctors! How could no one have noticed it for all those years?! No one saw it?! And now my daughter has the disease on top of it! I got my diagnosis at 39 years old, six years ago, when my daughter’s spots were diagnosed.* (Interview 20)

### From phenotypic diagnosis to genetic testing

In the vast majority of the respondents, the diagnosis of the disease was based on clinical symptoms, with only a few undergoing genetic testing for neurofibromatosis. However, this is not surprising considering that the largest group of respondents received their NF1 diagnosis in the 1990s, when phenotypic diagnosis was the standard diagnostic approach. The lack of access to genetic testing at that time hindered a full understanding of the disease and its hereditary nature. The nonspecific symptoms of NF1 were often misinterpreted, and the awareness of primary care physicians regarding rare diseases was alarmingly low. Similarly, access to knowledge about NF-related syndromes and to genetic testing itself was limited. At that time, the diagnosis of NF1 was mainly based on medical documentation resulting from numerous specialist consultations, without the support of molecular testing.

*Yes, the diagnosis was in 1996 – Recklinghausen’s disease. I had a neurological consultation. An MRI revealed a tumour in my head, which confirmed that it was indeed Recklinghausen’s disease. (…) Back then, genetic testing wasn’t done; the diagnosis was confirmed based on medical documentation.* (Interview 32)

*If someone, like me, has a few spots but no tumours and is only 4 or 5 years old, it’s hard to definitively say whether it’s the disease. Nowadays, genetic tests are done, but going back 25 years, such tests didn’t exist.* (Interview 45)

After years of consultative and exploratory examinations, the respondents needed an immediate and unequivocal diagnosis. They saw genetic testing as the key to this certainty. Even if the NF1 diagnosis had been made earlier, the respondents felt the need to confirm it through genetic testing. This was important for the identity of the affected individual. Nevertheless, genetic tests do not always provide a clear answer. Despite full clinical symptoms, genetic confirmation was sometimes lacking, highlighting the complexity and challenges of molecular diagnostics. A negative genetic test result does not invalidate an NF1 diagnosis established based on imaging studies and clinical symptoms. Diagnosis based on genetic testing is a prolonged process of discovery and testing rather than a one-time event.

*I knew I had it; the genetic test was more like confirmation and an opening to further examinations. (…) The geneticist told me right away that I would definitely have the disease, but they needed to do the test just to be sure. So I had the genetic test done, and after some time I received the results – of course, positive.* (Interview 67)

*Professor* [doctor’s name]*, based on the symptoms and a biopsy of the tumour on my face, diagnosed that it might be a mild form of neurofibromatosis type 1. She said I could undergo genetic testing for it. (…) That prompted me to start the process of identifying the disease. I went for genetic testing based on blood samples, which were sent to a specialized clinic in the United States that handles this kind of testing. However, based on the samples they received, they could not confirm the disease. They were unable to give a clear diagnosis. (…) I intended to continue the genetic testing, but of course, everything was paid for out of my own pocket.* (Interview 76)

*The genotypes were marked, but the test didn’t detect NF1. I even talked to the doctor, and she said that it’s a difficult gene to catch. But I have all the clinical symptoms.* (Interview 37)

### (Un)expected and (un)wanted diagnosis

A quick and short diagnostic pathway applies to symptoms that are easily noticeable but deeply distressing for the patient and their family, requiring immediate action. Heart problems, fatigue, fainting, shortness of breath, and persistent headaches become the starting point for an NF1 diagnosis. It is the subjective experience of the body – not visible skin changes – that triggers the medical diagnostic process. The NF1 diagnosis often occurs at moments least expected by the patient. Atypical symptoms, seemingly unrelated to neurofibromatosis, ultimately lead to the diagnosis being made.

*In ninety-seven, I suddenly started snoring and choking. They began examining my heart. It turned out I had a tumour in the left mediastinum, causing pressure. At first, the doctors suspected it was a malignant growth, and my attending doctor sent me to* [city name]*. After conducting all the tests, including a biopsy, they administered chemotherapy, but it didn’t help. The doctors said they could do no more and sent me to* [name of a specialized paediatric treatment centre in a large city]*. That’s where they removed the tumour. It was shocking that someone so young had a tumour the size of two surgeons’ fists. I was diagnosed with NF1 then and have been treated there ever since.* (Interview 16)

*It happened in childhood. I began having heart problems, started fainting, was constantly tired, and couldn’t run. I went with my mom to see a doctor, a cardiologist, who diagnosed me with a mitral valve prolapse. Later, I was referred to* [name of a large, specialized paediatric treatment centre]*, where NF1 was diagnosed.* (Interview 14)

Another circumstance in which a medical diagnosis of NF1 is made is a sudden hospital admission due to an acute illness, accident, or accidental injury that necessitates imaging studies (CT scan, MRI). In such cases, the diagnosis is not the result of planned clinical or genetic testing but a consequence of a physical incident. The onset of the diagnosis is unclear and nonspecific. The NF1 diagnosis is made somewhat “incidentally” during the investigation of another health problem requiring hospitalization.

*When I was 18, I was hit by a car. (…) And in the hospital where I ended up, there was no equipment. It was a small hospital. Then my parents, through connections, arranged for me to stay at* [name of a large hospital in the regional city]*. I had an MRI of my head done there, and luckily, by a stroke of fate, a doctor who was familiar with this disease recognized that I have NF1.* (Interview 17)

*My parents found out about the disease when I was five years old. (…) One day, I got pneumonia and ended up in the hospital, and the paediatrician simply said it was Recklinghausen’s disease.* (Interview 12)

The diagnosis also sometimes appears as an incidental result of cosmetic procedures. The entry into the patient role does not occur because of medical symptoms – since neurofibromas are not defined as such here – but because of a cosmetic defect. A visit to a doctor, most often a dermatologist or surgeon, to remove neurofibromas scattered across the body often becomes a turning point.

*I went to a dermatologist to have some nodules removed. I was in my thirties at the time. The dermatologist kind of scared me because she said she wouldn’t remove them and that it would be best for me to get tested. The dermatologist told me it was probably NF1. So, I went to a neurologist, who indeed confirmed that it was the disease.* (Interview 19)

Situations leading to a rapid NF1 diagnosis often involve contact with an experienced specialist, frequently employed at a reputable centre, who breaks through the previous diagnostic deadlock. A high level of diagnostic expertise among doctors increases the patient’s sense of security and confidence in the disease diagnosis. The following accounts from the respondents highlight the value of access to qualified specialist doctors and reference centres, which play a key role in establishing the diagnosis of this rare disease.

*The doctors at* [name of a specialized centre] *just knew right away what it was. I ended up there when I was four years old (…). The professor immediately recognized the disease at first glance. He didn’t need any genetic tests or anything else.* (Interview 9)

*For a long time, I was treated typically for vision issues – covering the eye, using a darkroom, and so on. Later, my ophthalmologist went on maternity leave, and I was assigned to another eye doctor who noticed Lisch nodules on my eye, referred me for further tests, and that’s how I ended up getting a CT scan that confirmed neurofibromatosis.* (Interview 52)

## Discussion

The findings of this study highlight the complexity and multidimensionality of the diagnostic experience for individuals with NF1. Participants attributed diagnostic delays to both systemic shortcomings, such as limited access to specialists, insufficient physician training, and regional disparities, as well as lay and clinical misinterpretation of early symptoms. Families played a dual role, at times facilitating diagnosis through active advocacy, but also occasionally contributing to delays through symptom minimization or denial. These dynamics were compounded by emotional burdens linked to the genetic nature of NF1, leading to frustration, isolation, and a sense of abandonment. Hence, the accounts presented in this study highlight several important aspects of the diagnostic experience and point to a pressing need for heightened awareness, earlier identification, and more cohesive models of care.

Firstly, one of the most significant obstacles to early diagnosis was the pervasive normalization of symptoms. Even when hallmark features, such as CALMs or subcutaneous nodules, were present, they were often dismissed by both families and clinicians as non-threatening aesthetic traits. This pattern appears to reflect culturally shaped lay concepts of health, disease, and the body, in which minor skin changes or anomalies are perceived as typical variations rather than signs warranting medical attention (Orfei et al., 2008; Snell-Rood and Carpenter-Song, 2018; Faye et al., 2024). This is consistent with previous findings showing that lay interpretations often contribute to the minimization or disregard of symptoms, leading individuals to perceive them as temporary, benign, or stress-related rather than as potential medical concerns. Such interpretations may postpone help-seeking and thereby delay diagnosis (Gannik and Jespersen, 1984). Similar normalizing strategies were also observed in a study of families affected by lysosomal storage disorders, where ambiguous early signs were frequently interpreted as benign or “part of the child’s nature,” reinforcing diagnostic delays (Awada and Holcik, 2023).

Given that many respondents had no family history or prior knowledge of NF1, participants’ accounts illustrate how insufficient contextual understanding can contribute to the misinterpretation of bodily signs. This is in line with previous studies showing that cultural health beliefs and social norms influence how people make sense of their symptoms, often reinforcing assumptions of harmlessness (Eisenberg, 1977; Orfei et al., 2008; Zurynski et al., 2017; Au et al., 2022; Karwacki, 2025). For example, chronic back pain was commonly attributed to poor posture – a “weak” or “crooked” spine instead of an underlying condition (Dowrick et al., 2004). Similarly, research shows that patients with rare conditions often overlook subtle or nonspecific symptoms (e.g., chronic fatigue), attributing them to aging, stress, or work demands, and typically seek medical attention only once the changes become noticeable to others (Phillips et al., 2024). It was also noted that families frequently framed subtle symptoms within everyday explanatory models, such as fatigue resulting from school stress, highlighting a broader phenomenon across rare conditions where everyday reasoning delays specialist referral (Awada and Holcik, 2023).

Importantly, participants’ accounts suggest that clinicians may contribute to these diagnostic delays. Many participants reported that their concerns were minimized or explained away as psychological or lifestyle-related, for instance, resulting from stress or aging (olde Hartman et al., 2009; Phillips et al., 2024). Prior studies indicate that general practitioners frequently interpret ambiguous symptoms as falling within a “normal range”, requiring no further action (Dowrick et al., 2004; olde Hartman et al., 2009; Dures et al., 2019). In some cases, the symptoms were reduced to isolated interpretations. Nonetheless, previous studies showed that beyond normalization, physicians often display a tendency toward particularization, that is, the tendency to reduce symptoms to a single organ system (e.g., fatigue attributed to depression, menopause, or iron deficiency; respiratory issues to asthma or infection), while overlooking the possibility of a multisystem disorder (Phillips et al., 2024). Others highlight the phenomenon of medical gaslighting, where physicians dismiss or undermine patients’ experiences, discouraging symptom reporting and contributing to lengthy diagnostic odysseys and systemic mistrust (Au et al., 2022; Domaradzki and Walkowiak, 2024). Similar patterns have also been documented in other rare diseases, where fragmented medical encounters and limited disease-specific knowledge created cycles of repeated referrals and deepened families’ uncertainty, dynamics strongly resonant with the diagnostic odyssey described by individuals with NF1 (Awada and Holcik, 2023).

Secondly, the participants described multiple systemic challenges, including a lack of diagnostic guidelines, underdeveloped specialist networks, and logistical or bureaucratic hurdles. In our data, diagnoses were often missed or misclassified, consistent with what has been reported in earlier studies (Cnossen et al., 1997; Bauskis et al., 2022; García-Martínez and Hernández-Martín, 2023; Dyer et al., 2024). For NF1, diagnostic delays typically range from 4 to 6 years, even though characteristic clinical signs often appear much earlier in childhood (García-Martínez and Hernández-Martín, 2023). Children with no familial precedent were particularly susceptible to delays as their symptoms were often mistaken for harmless traits (Cnossen et al., 1997; García-Martínez and Hernández-Martín, 2023). In specific cases, such as pregnancy, skin changes were misattributed to hormonal shifts, adding further complexity to the diagnosis (Dyer et al., 2024). Moreover, those with milder presentations typically faced the longest delays. In nearly all cases, definitive diagnoses were confirmed in specialist centres, emphasizing the need for improved screening protocols and streamlined care pathways (Evans et al., 2025; Karwacki, 2025).

Participants described diagnostic delays as having wide-ranging impacts, extending beyond the clinical implications. The respondents reported psychological strain, disrupted family planning, and adverse effects on their overall quality of life (Crawford et al., 2015; Rietman et al., 2018; Gonzalez et al., 2024; Kowal and Domaradzki, 2025). The individuals diagnosed later in life, especially after becoming parents, often expressed feelings of regret, disappointment, and disillusionment with the healthcare system. Many arrived at a diagnosis only after sustained personal effort, private medical consultations, or chance events such as accidents or a child’s diagnosis. These experiences highlight a need for more coordinated diagnostic pathways for NF1 and enhancing healthcare provider training (Evans et al., 2025; Karwacki, 2025).

The participants also drew attention to class- and geography-based barriers in access to diagnostic services. Many highlighted how regional disparities, the absence of diagnostic infrastructure in the 1990s, a lack of reference centres outside major cities, and the high cost of private testing further affected the timing and quality of the diagnosis. The lack of local diagnostic services and high costs of private testing were persistent barriers to equitable care (Anderson et al., 2013; Teutsch et al., 2023; Domaradzki and Walkowiak, 2024).

Echoing prior research, many respondents attributed diagnostic delays to what they perceived as limited preparedness among general practitioners, noting gaps in training on rare genetic conditions, particularly within primary care (Vandeborne et al., 2019; Ramalle-Gómara et al., 2020; Walkowiak and Domaradzki, 2021; Zhang et al., 2022; Flores et al., 2022; Walkowiak et al., 2022).

Thirdly, due to a lack of institutional guidance and a broader sense of inadequate systemic support, which left them feeling frustrated, disoriented, insecure, and isolated, families played a complex and at times contradictory role in the diagnostic journey (Currie and Szabo, 2019b; Domaradzki and Walkowiak, 2024). While many respondents, especially mothers, stepped into leadership roles, navigating the healthcare system and advocating for services, others inadvertently contributed to diagnostic delays by overlooking, downplaying, or not disclosing early symptoms. Without institutional support, families were often left to manage on their own (Currie and Szabo, 2019b; 2019a; Gómez-Zúñiga et al., 2021; Domaradzki and Walkowiak, 2024). Participants described this self-reliance as resourceful, though often associated with personal strain. At the same time, this study supports previous findings showing that tensions often arose when patients or caregivers possessed greater condition-specific knowledge than healthcare professionals (Budych et al., 2012; Witt et al., 2021; Domaradzki and Walkowiak, 2024).

However, this dynamic was not uniform, as in some cases, denial stemmed from emotional conflict: mothers suppressed suspicions or avoided confronting symptoms resulting from fear, shame, or guilt (Kowal and Skrzypek, 2024; Currie and Szabo, n.d.). Such dynamics, shaped by stigma or taboo, have also been observed in the context of other inherited and stigmatized disorders, particularly in mental health (Murphy et al., 2017; Dobener et al., 2022). This emotional ambivalence mirrors findings from other rare-disease contexts, in which caregivers simultaneously occupy positions of vigilance, denial, and self-protection, reflecting the relational and moral strain inherent in managing genetic or lifelong conditions (Awada and Holcik, 2023). Thus, this ambivalence underscores the family’s dual role in the diagnostic process and reflects not only informational or systemic deficiencies, but also the emotional complexity and moral weight of managing a genetic diagnosis within the family.

Finally, participants frequently described advocating for themselves or their children within a fragmented healthcare system, independently seeking information, consultations, and specialists. In many cases, the diagnosis of NF1 was not the result of a structured and coordinated medical pathway but rather stemmed from the patient’s persistence, incidental medical events (such as accidents), or the diagnosis of a child. While these actions demonstrate a form of *epistemic empowerment*, understood as patients’ growing ability to acquire, use, and assert condition-specific knowledge within medical encounters (Bogaert, 2021), they also reflect the psychological burden of navigating a fragmented system.

Moreover, the randomness of diagnosis, often occurring after years of fragmented medical encounters, was not always experienced as empowering. For some, the unpredictability and exhaustion of the diagnostic process were sources of distress rather than relief. Participants described experiencing what they themselves referred to as “diagnostic fatigue”, a state of emotional and physical depletion resulting from extended efforts to seek validation and care (Cnossen et al., 1997; García-Martínez and Hernández-Martín, 2023; Dyer et al., 2024; Bauskis et al., 2022).

## Limitations

Although this is one of the first studies on the diagnostic experiences of individuals with NF1 in Poland, it has several limitations. While the sample of 93 participants is large for a qualitative study, it does not represent the entire NF1 population in the country, especially given the absence of a national rare disease registry. The qualitative and locally focused nature of the research limits its generalizability and comparability with findings from other countries. The participants were recruited from a single hospital through two physicians, potentially excluding individuals from other regions or healthcare settings. In addition, individuals with advanced NF1 were underrepresented, and most of the respondents were women, which may limit the transferability of findings to men or to those in later stages of the disease. The use of phone and online interviews–necessitated by the COVID-19 pandemic–may have prevented the observation of non-verbal cues, which could have enriched the contextual understanding and limited the researchers’ ability to capture some aspects of participants’ embodied experiences. On the other hand, many participants indicated that this form of communication increased their sense of comfort due to the bodily changes caused by NF1, and several explicitly stated that they would not have agreed to participate in a face-to-face interview.

Moreover, because many participants reconstructed early-life or childhood experiences retrospectively, their accounts may be subject to recall bias, potentially shaping how they interpret past events. The gender imbalance in the sample, with women substantially overrepresented, may also influence the thematic emphasis, especially in areas related to caregiving and family dynamics. Recruitment through specialist clinics may have introduced a selection bias, as individuals without access to specialised centres or those less engaged with healthcare services might be underrepresented.

Despite these limitations, several strengths of the study should be acknowledged. Most importantly, given the scarcity of prior research, this study helps fill a significant gap in the literature regarding the diagnostic experiences of individuals with NF1 in Poland. Moreover, by giving voice to patients, it offers valuable insights into a neglected area, laying the groundwork for future research and the development of targeted support strategies. Finally, the opportunity for individuals with NF1 to share their personal stories may have had therapeutic value.

## Conclusion

This study reveals that the diagnostic process for NF1 in Poland is long, fragmented, and burdened with uncertainty. Rather than a discrete clinical event, diagnosis emerges as a drawn-out, multi-stage journey shaped by medical, social, and emotional factors.

The findings emphasize that the absence of early recognition, despite the presence of visible symptoms, has serious consequences for timely medical intervention, emotional wellbeing, and informed reproductive choices. While the role of family, especially mothers, was often crucial in pursuing a diagnosis, the lack of systemic support frequently forced patients and caregivers to act as informal coordinators of their own diagnostic journeys.

In light of these findings, several practical steps can be taken to improve early recognition, reduce diagnostic delays, as well as support individuals and families affected by NF1:

1. Develop NF1-specific training modules for general practitioners and pediatricians, particularly as part of continuing medical education (CME). They should focus on early cutaneous symptoms (e.g., CALMs) and raise awareness of the heterogeneity and progression of NF1.
2. Implement routine dermatological screening protocols in primary care, paediatric clinics, and, where applicable, school health programs, particularly for infants and children with multiple pigmented skin lesions, to support early identification and referral of potential NF1 cases.
3. Establish standardized, interdisciplinary referral pathways, linking primary care with dermatology, neurology, oncology, and genetics, to streamline diagnostic procedures and prevent the fragmentation of care.
4. Expand regional access to genetic counselling and testing by integrating these services into local hospitals, thereby minimizing geographic and financial barriers to molecular diagnostics.
5. Develop accessible, patient-centred educational materials, tailored to different stages of life and diagnosis, to empower individuals and reduce the uncertainty and isolation often experienced in the pre- and post-diagnostic phases.
6. Introduce dedicated psychosocial support services, including psychological counselling and peer support, for individuals with NF1 and their families, both at the time of diagnosis and throughout their medical journey.
7. Establish a national NF1 registry and integrated care frameworks, and support the development or strengthening of organisations and collaborative networks that engage families, researchers, and medical professionals. Such structures can coordinate services, monitor prevalence, enhance public and professional awareness, and help ensure equitable access to timely diagnosis and treatment across Poland.

Together, these recommendations outline practical steps that could significantly improve the diagnostic experience for people with NF1. Strengthening early recognition, specialist coordination, and family- and patient-oriented support systems has the potential to reduce uncertainty, shorten diagnostic delays, and enhance the quality of life for individuals and families affected by NF1. Implementing these measures would help create a more responsive, equitable, and patient-centred model of care.

## Data Availability

The original contributions presented in the study are included in the article/Supplementary Material, further inquiries can be directed to the corresponding author.
