# Supplementary material for: (Not) “just your nature”. Diagnostic journeys of adults with neurofibromatosis type 1 in Poland
Source: Front Genet. 2026 Jan 7;16:1713304. doi: 10.3389/fgene.2025.1713304 (PMC12818789; doi:10.3389/fgene.2025.1713304)
Supplement: Supplementary file 1 [file Table1.docx]

**FILE 1. COREQ: 32-ITEM CHECKLIST**

| **No** | **Item** | **Guide questions/description** | **The authors’ responses to the manuscript** |
| --- | --- | --- | --- |
| **Domain 1: Research team and reflexivity** |  |  |  |
| 1 | Interviewer/facilitator | Which author/s conducted the interview or focus group? | KK – first author of the text |
| 2 | Credentials | What were the researcher’s credentials? E.g. PhD, MD | Doctor of Humanities in Sociology |
| 3 | Occupation | What was their occupation at the time of the study? | Sociologist, academic teacher, research staff |
| 4 | Gender | Was the researcher male or female? | Woman |
| 5 | Experience and training | What experience or training did the researcher have? | The interviewer was the first author of this article, a sociologist by profession. Katarzyna Kowal holds a PhD in sociology with specializations in (1) the sociology of health, illness, and medicine; (2) body sociology; and (3) qualitative sociology. For the past 25 years, she has conducted sociological research within the interactional and interpretive paradigm. |
| **Relationship with participants** |  |  |  |
| 6 | Relationship established | Was a relationship established prior to study commencement? | None of the participants had any relationship with the researcher, and there were no interactions between them before the start of the study. |
| 7 | Participant's knowledge of the interviewer | What did the participants know about the researcher? e.g. personal goals, reasons for doing the research | The researcher had no personal experiences or particular reasons for engaging in this research topic. This information was communicated to all potential participants before the study. |
| 8 | Interviewer characteristics | What characteristics were reported about the interviewer/facilitator? e.g., Bias, assumptions, reasons, and interests in the research topic | Potential participants were provided with information about the researcher, including her full name, the name of the university where she works, and detailed information about the faculty and department in which she is employed, as well as her academic position. The participants were also informed that the researcher’s interest in the study topic was purely professional. |
| **Domain 2: Study design** |  |  |  |
| **Theoretical framework** |  |  |  |
| 9 | Methodological orientation and Theory | What methodological orientation was stated to underpin the study? e.g., grounded theory, discourse analysis, ethnography, phenomenology, content analysis | Reflexive Thematic Analysis (RTA) according to the concept of V. Braun and V. Clarke |
| **Participant selection** |  |  |  |
| 10 | Sampling | How were participants selected? e.g. purposive, convenience, consecutive, snowball | The sampling was purposive. The following inclusion criteria were used: 1) being over 18 years old, 2) having a medically confirmed diagnosis of NF1, 3) speaking Polish, 4) having access to the Internet, 5) being willing to participate, and 6) providing voluntary informed consent to participate. |
| 11 | Method of approach | How were participants approached? e.g., face-to-face, telephone, mail, email | Participants contacted the researcher themselves, either by phone or by email. |
| 12 | Sample size | How many participants were in the study? | 93 |
| 13 | Non-participation | How many people refused to participate or dropped out? Reasons? | Two participants withdrew from the study, indicating a lack of time as the reason. |
| **Setting** |  |  |  |
| 14 | Setting of data collection | Where was the data collected? e.g., home, clinic, workplace | As the interviews were conducted by phone or online, most respondents took part from their own homes, creating a comfortable and safe atmosphere for open discussion. |
| 15 | Presence of non-participants | Was anyone else present besides the participants and researchers? | Care was taken to ensure that no other individuals were present in the environment of either the researcher or the participants during the interviews. |
| 16 | Description of sample | What are the important characteristics of the sample? e.g., demographic data, date | Gender, age, age at diagnosis, education, marital status, place of residence – See Table 1. Sociodemographic characteristics of participants |
| **Data collection** |  |  |  |
| 17 | Interview guide | Were questions, prompts, and guides provided by the authors? Was it pilot tested? | The first author prepared an in-depth interview guide (see Supplementary File 1). In line with common practice in qualitative research, a “soft pilot” was conducted, during which the questions were tested with one or two individuals to refine the guide without undertaking a formal pilot study. |
| 18 | Repeat interviews | Were repeat interviews carried out? If yes, how many? | Given the depth and richness of the interviews, as well as the iterative nature of the analytic process, we were able to fully interpret and develop the themes without the need for follow-up interviews. Minor ambiguities that naturally arise in qualitative interviewing were resolved through careful contextual analysis, cross-case comparison, and analytic memo-writing. |
| 19 | Audio/visual recording | Did the research use audio or visual recording to collect the data? | The interviews were audio-recorded with participants’ consent. In total, 134 hours, 26 minutes, and 35 seconds of audio material were collected. |
| 20 | Field notes | Were field notes made during and/or after the interview or focus group? | After each interview, the researcher prepared field notes, so-called “memos,” which included: the researcher’s reflections, preliminary interpretations, contextual data, suggestions for subsequent interviews, information on encountered difficulties and problems, and general impressions from the interview. |
| 21 | Duration | What was the duration of the interviews or focus group? | Each session lasted around 90 minutes. |
| 22 | Data saturation | Was data saturation discussed? | In the applied Reflexive Thematic Analysis (RTA) approach following Braun and Clarke, the emphasis was placed not on the concept of “data saturation” but on achieving depth of meaning and interpretive clarity (Braun & Clarke, 2021b, 2023). |
| 23 | Transcripts returned | Were transcripts returned to participants for comment and/or correction? | No, this was not necessary. |
| **Domain 3: Analysis and findings** |  |  |  |
| **Data analysis** |  |  |  |
| 24 | Number of data coders | How many data coders coded the data? | One researcher, KK, participated in the coding. |
| 25 | Description of the coding tree | Did the authors provide a description of the coding tree? | In line with the Reflexive Thematic Analysis approach, the authors did not construct a formal coding tree but presented the developed themes and their relationships in Table 2. |
| 26 | Derivation of themes | Were themes identified in advance or derived from the data? | In line with Braun and Clarke’s Reflexive Thematic Analysis (RTA), themes were not predetermined but developed through a reflexive and iterative process of engagement with the data. Rather than being simply identified, themes were actively constructed by the researchers through interpretive analysis. |
| 27 | Software | What software, if applicable, was used to manage the data? | All coding was performed manually, without the use of qualitative data analysis software, which aligns with the close and interpretive engagement emphasised in RTA. |
| 28 | Participant checking | Did participants provide feedback on the findings? | Yes. Feedback from patients who participated in the study indicated that their definitions and understandings of the NF1 experience were consistent with the authors’ interpretations. This feedback was obtained after the presentation of the study findings at the Neurofibromatosis Poland Association Symposium, held on November 26–27, 2022, in Warsaw. |
| **Reporting** |  |  |  |
| 29 | Quotations presented | Were participant quotations presented to illustrate the themes/findings? Was each quotation identified? e.g. participant number | Yes, the study findings were illustrated with multiple participant quotations, each identified by an interview number. |
| 30 | Data and findings are consistent | Was there consistency between the data presented and the findings? | Yes, the findings are well supported by the data, with participant quotations used to illustrate the identified themes. |
| 31 | Clarity of major themes | Were major themes clearly presented in the findings? | Yes, eight major themes were presented in the findings, each of which also served as a subsection within the Results section. |
| 32 | Clarity of minor themes | Is there a description of diverse cases or a discussion of minor themes? | In the presentation of the study findings, descriptions of diverse participant cases and minor variations were provided to illustrate the breadth of experiences. |
